# QTLs for Resistance to Major Rice Diseases Exacerbated by Global Warming: Brown Spot, Bacterial Seedling Rot, and Bacterial Grain Rot

**DOI:** 10.1186/s12284-016-0095-4

**Published:** 2016-05-13

**Authors:** Ritsuko Mizobuchi, Shuichi Fukuoka, Seiya Tsushima, Masahiro Yano, Hiroyuki Sato

**Affiliations:** National Institute of Agrobiological Sciences, 2-1-2 Kannondai, Tsukuba, Ibaraki 305-8602 Japan; National Institute for Agro-Environmental Sciences, 3-1-3 Kannondai, Ibaraki, 305-8604 Japan; NARO Institute of Crop Science (NICS), 2-1-18 Kannondai, Tsukuba, Ibaraki 305-8518 Japan; National Agriculture and Food Research Organization, Kyushu Okinawa Agricultural Research Center (NARO/KARC), 496 Izumi, Chikugo, Fukuoka 833-0041 Japan

**Keywords:** *Oryza sativa* L, Disease resistance, Brown spot, Seedling rot, Grain rot, Panicle blight, *Bipolaris oryzae*, *Burkholderia glumae*

## Abstract

In rice (*Oryza sativa* L.), damage from diseases such as brown spot, caused by *Bipolaris oryzae,* and bacterial seedling rot and bacterial grain rot, caused by *Burkholderia glumae*, has increased under global warming because the optimal temperature ranges for growth of these pathogens are relatively high (around 30 °C). Therefore, the need for cultivars carrying genes for resistance to these diseases is increasing to ensure sustainable rice production. In contrast to the situation for other important rice diseases such as blast and bacterial blight, no genes for complete resistance to brown spot, bacterial seedling rot or bacterial grain rot have yet been discovered. Thus, rice breeders have to use partial resistance, which is largely influenced by environmental conditions. Recent progress in molecular genetics and improvement of evaluation methods for disease resistance have facilitated detection of quantitative trait loci (QTLs) associated with resistance. In this review, we summarize the results of worldwide screening for cultivars with resistance to brown spot, bacterial seedling rot and bacterial grain rot and we discuss the identification of QTLs conferring resistance to these diseases in order to provide useful information for rice breeding programs.

## Introduction

Climate changes have widespread impacts on human life. The Intergovernmental Panel on Climate Change (IPCC) published the Fifth Assessment Report (AR5) in 2014 which provided a clear and up-to-date view of the current state of scientific knowledge relevant to climate change. According to this report, warming of the climate system is unequivocal, and many of the observed changes are unprecedented over decades to millennia (IPCC 2014). Specifically, the globally averaged combined land and ocean surface temperature data as calculated using a linear model show warming of 0.85 °C over the period 1880 to 2012 (IPCC 2014).

Among its many effects, global warming influences the distribution, incidence and severity of plant disease worldwide (Juroszek and von Tiedemann 2015). In rice (*Oryza sativa* L.), many pests and the pathogens they transmit are influenced by global warming. For example, a geographical shift in the prevalence of rice stripe virus disease, transmitted by the small brown planthopper, is predicted to result from global warming in Japan (Yamamura and Yokozawa 2002). In addition, several pathogens are expected to be directly influenced by global warming. Among these are pathogens that cause agriculturally important diseases such as sheath blight, caused by *Rhizoctonia solani*, brown spot (BS), caused by *Bipolaris oryzae*, and bacterial seedling rot (BSR) and bacterial grain rot (BGR), both caused by *Burkholderia glumae*, because the optimal temperature ranges for growth of these pathogens are relatively high (around 30 °C). Sheath blight is considered as one of the three major diseases of rice (along with blast and bacterial blight) (Zou et al. 2000; Hu et al. 2008; Liu et al. 2010), and many studies have been done to enhance resistance to sheath blight. A review covering QTLs for resistance to sheath blight has been published recently (Zeng et al. 2015), so this topic is not covered here.

Compared with the extensive research on sheath blight, less research has been done to enhance resistance to BS, BSR and BGR. However, reports of damage caused by these diseases are increasing under global warming conditions because, as noted above, their optimal growth temperatures are relatively high (Ham et al. 2011b; Savary et al. 2011). The optimal temperatures for conidial germination and hyphal germination of *Bipolaris oryzae* are 25–30 °C and 27–30 °C, respectively (Barnwal et al. 2013), and the optimal temperature range for the growth of *Burkholderia glumae* is 30–35 °C (Kurita et al. 1964; Tsushima et al. 1986).

According to a recent review (Barnwal et al. 2013), BS is currently regarded as a serious rice disease worldwide. BS was identified in 1892 in Japan (Ohata 1989) and can often considerably decrease grain yield and quality. For example, this disease was the main cause of the Bengal famine of 1943 (Padmanabhan 1973). More recently, BS has been also reported in Brazil (Schwanck et al. 2015) and in East and Southeast Asian countries (Savary et al. 2000b) and India (Reddy et al. 2010), and the reported yield losses vary from 4 to 52 % (Savary et al. 2000a; Barnwal et al. 2013). The damage caused by BS generally becomes noticeable when rice is grown in nutritionally deficient or otherwise unfavorable soil conditions (Katara et al. 2010). Yield losses due to BS have been estimated to range between 16 % and 43 % on Histosols, which are Si-deficient (Datnoff et al. 1991). In 2013, the epidemic area of BS in Japan covered 187,714 ha, making it the fourth most prevalent rice disease of that year (after sheath blight, leaf blast and neck blast) (JPPA 2014).

BGR and BSR are also increasingly important diseases in global rice production (Ham et al. 2011b). The term ‘bacterial panicle blight’ is also used to refer to BGR, mainly in the United States and Latin American countries (Ham et al. 2011b). The causal organism of these diseases, *Burkholderia glumae*, was identified in 1955 in Japan (Goto and Ohata 1956; Kurita and Tabei 1967; Uematsu et al. 1976; Goto et al. 1987) and has also been reported in the United States (Nandakumar et al. 2009), East and South Asia (Chien and Chang 1987; Trung et al. 1993; Cottyn et al. 1996a; Cottyn et al. 1996b; Jeong et al. 2003; Luo et al. 2007), Latin America (Zeigler and Alvarez 1989; Nandakumar et al. 2007b) and South Africa (Zhou 2014). In 2013, the epidemic area of BGR in Japan covered 69,799 ha, making it the sixth most prevalent disease (following leaf stripe, the fifth most prevalent disease) of that year (JPPA 2014). In the United States, *Burkholderia glumae* has been identified as the major causal agent of BGR (Shahjahan et al. 2000; Nandakumar et al. 2005; Nandakumar et al. 2009). In the southern United States, yield losses caused by outbreaks of BGR in rice fields in Arkansas were up to 50 % in 2010 and 2011; significant losses caused by this disease were also experienced in other recent years (Shahjahan et al. 2000; Nandakumar et al. 2009; Ham et al. 2011a; Ham et al. 2011b; Zhou et al. 2011; Wamishe et al. 2014).

As illustrated above, it is necessary to breed rice cultivars with resistance to BS, BSR and BGR. To date, several research groups have successfully identified cultivars with some level of resistance to these diseases. However, despite extensive efforts, no single genes or QTLs for complete resistance to BS, BSR or BGR have been discovered. Recent progress in molecular genetics and improvement of evaluation methods for disease resistance have allowed us to identify several quantitative trait loci (QTLs) to enhance resistance to these pathogens. In this review, we focus on host plant resistance to BS, BSR and BGR. We summarize the research on QTLs related to resistance to these diseases and discuss the potential utility of these QTLs for rice breeding.

## Review

### Screening cultivars for brown spot (BS) resistance

Several studies have been conducted to screen cultivars for BS resistance (Table 1). Rice plants are highly susceptible to BS at the seedling stage and between the booting and flowering stages (Chakrabarti 2001). In 1930, ‘Kutto-urupe’, which was described as a Korean native cultivar, was found to be more susceptible than other cultivars by field observation without artificial inoculation (Nagai and Hara 1930); this was the first report that showed varietal differences in BS resistance levels. Later, ‘Mubo-Aikoku’ was found to be resistant to BS by seedling inoculation tests in the United States (Adair 1941). Yoshii and Matsumoto applied spray inoculation to plants of different stages (seedling, tillering, booting and flowering) and demonstrated that two cultivars (‘Tetep’ and ‘Ginnen’) were resistant and another six cultivars, including ‘Tadukan’, were moderately resistant (Yoshii and Matsumoto 1951). In Egypt, ‘Pi1’ and ‘YNA282’ were categorized as resistant (Balal et al. 1979).

**Table 1:** Previous reports of screening cultivars and breeding lines for brown spot (BS) resistance

Year	Country^a^	Resistant cultivars and breeding lines	Number of screened cultivars or lines	Evaluation method	Reference(s)
1930	Japan	(Kutto-urupe identified as a susceptible cultivar)	-	Field observation without artificial inoculation	Nagai and Hara (1930)
1941	USA	Mubo-Aikoku	16	Inoculation of seedlings	Adair (1941)
1951	Japan	Resistant: Tetep, Ginnen	20	Inoculation of different growth stages (seedling, tillering, booting and flowering)	Yoshii and Matsumoto (1951)
		Moderately resistant: Tadukan, Bomba, Jamaica, Louisiana non-beard, Choukakou, Shimoushaku			
1962	Japan	Kairyo-Aikoku, Ginbozu and 20 breeding lines	45	Field observation between different soils (K-deficient field, sandy loam and peat soil) over 11 years	Yasumasa et al. (1962)
1974	Japan	Tadukan, Tetep, Jamaica, Pi No.1, Kararato, Choukakou, Usen, Ginnen	80	Inoculation of seedlings	Ohata and Kubo (1974), Ohata (1989)
1979	Egypt	Pi1, YNA282	5	Inoculation at four stages (seedling, maximum tillering, maximum heading and milk-ripe stage)	Balal et al. (1979)
1985	India	CH45	-	Field observation without artificial inoculation	Misra (1985)
1986	USA	Dawn, Taichung Native-1, Tetep	11	Inoculation of 45-day-old-plants	Eruotor (1986)
1994	USA	Katy, Experimental Line 1^b^	10	Field observation without artificial inoculation (Si-deficient soil, Histosol)	Deren et al. (1994)
1995	India	Jhllidhan (HRC 703), Kalamdani (HRC 711), Tulsimanjari (HRC 719), Bankuiya (HRC 729), Marto (NIC 105696)	183	Field observation without artificial inoculation over 4 years	Shukla et al. (1995)
2004	Bangladesh	Line 139^c^	33	Field observation without artificial inoculation over 2 years	Hossain et al. (2004)
2005	India	Resistant: Khazar, Teqing, Tarommolaii, IR6, Chhomrong, Govind, UPR191-66, ASD18, R644, Yuanjing7, Xu-Xiangzan, BG90-2, TKM9, Guang122	124	Inoculation of 70-day-old-plants after transplanting	Satija et al. (2005)
		Moderately resistant: RASI, IR64, CR203, IR50, BG304, Lemont, Phalguna, PR111			
2006	India	CR 100117, CR 100140, CR 100142, CR100142A (all are *Oryza nivara *germplasm)	150	Inoculation of 90-day-old plants (all accessions were O. nivara)	Goel et al. (2006)
2006	India	(PR116, PR114 and PR106 detected as susceptible cultivars)	9	Field observation without artificial inoculation over 3 years	Pannu et al. (2006)

^a^Country where experiment was conducted

^b^Experimental Line 1 is an advanced line from the breeding program in Arkansas

^c^A breeding line

As summarized by Barnwal et al. (Barnwal et al. 2013), damage by BS has long been associated with soil fertility (Ou 1985; Ohata 1989). Moreover, the effects of soil nutrients depend on the redox potential of the soil in fields containing sandy loam and peat soil (Ou 1985; Ohata 1989; Barnwal et al. 2013). In Japan, the damage by BS was found to be quite severe on low-K soil (Ono 1953), sandy loam and peat soil (Ohata 1989). Therefore, screening was conducted in fields having different soil conditions (K-deficient field, sandy loam and peat soil) over 11 years, and two cultivars and 20 breeding lines were found to be resistant (Yasumasa et al. 1962). BS was also reported to be severe on low-Si soils (Datnoff et al. 1991; Datnoff et al. 1997), and the effect of Si on resistance to BS was ascertained recently by using a Si-transporter mutant (Dallagnol et al. 2014). By evaluating cultivars and breeding lines on Si-deficient soil, two were found to be resistant to BS (Deren et al. 1994). In a screen of 80 cultivars in 1974, eight cultivars including ‘Tadukan’ were found to be resistant to many strains (Ohata and Kubo 1974; Ohata 1989). Among these cultivars, ‘Tadukan’, ‘Tetep’, ‘Choukakou’ and ‘Ginnen’ were categorized as resistant by two research groups independently (Yoshii and Matsumoto 1951; Ohata and Kubo 1974; Ohata 1989). ‘Tetep’ was also categorized as resistant by another research group of the United States (Eruotor 1986).

Until the 20th century, most reports of BS were from Japan, but more recent reports from Bangladesh and India suggest that the damage by BS in South Asia has become serious (Chakrabarti 2001). In Bangladesh, ‘Line 139’ was reported to be resistant by field evaluation (Hossain et al. 2004). In three independent studies in India, more than 183 cultivars in total were screened and 28 showed partial resistance to BS (Misra 1985; Shukla et al. 1995; Satija et al. 2005). By field observation without artificial inoculation during 3 years in different districts in Punjab state of North India, three cultivars were categorized as susceptible (Pannu et al. 2006). Goel et al. (2006) evaluated 150 accessions of *Oryza nivara* (wild rice), and found that four were resistant to BS.

### Analysis of QTLs for resistance to brown spot (BS)

Several cultivars that have been categorized as resistant did not show complete resistance (immunity) to BS (Sreedharan and Menon 1974). Some varieties such as ‘Tadukan’ and ‘Tetep’ showed quantitative resistance to BS (Yoshii and Matsumoto 1951; Ohata and Kubo 1974; Eruotor 1986; Ohata 1989). The first QTL analysis for resistance to BS was conducted in 2008 (Sato et al. 2008a) (Table 2). In that study, a set of recombinant inbred lines (RILs) derived from a cross between ‘Tadukan’ (resistant) and ‘Hinohikari’ (susceptible) was used to identify QTLs conferring resistance to BS at the seedling stage in a greenhouse screen. Three QTLs (*qBS2*, *qBS9* and *qBS11*) were detected on chromosomes 2, 9 and 11, respectively. Among them, *qBS11* had the highest logarithm of odds (LOD) score (LOD = 5.11 in CIM (composite interval mapping) and 2.82 in IM (interval mapping)) and could be considered a major QTL. The ‘Tadukan’ allele explained 11.9 to 15.3 % of the phenotypic variation in the F_5_ population. The *qBS9* allele from ‘Tadukan’ could explain 9.7 to 12.9 % of the phenotypic variation, while the *qBS2* allele from ‘Hinohikari’ could account for 11.1 % of the total phenotypic variation.

**Table 2:** QTLs for brown spot (BS) resistance

Chromosome	QTL^a^	Source of resistance allele	Materials used for QTL analysis^b^	Phenotyping method	Reference
1	*qBSfR1*	Tadukan	110 RILs (Tadukan (R) and Hinohikari (S))	Field evaluation at 113 days after transplanting by promoting disease with inoculated spreader	Sato et al. (2015)
2	*BSq2.1v&i*	IR62266-42-6-2	154 DH lines (CT9993-5-10-1 M (R) and IR62266-42-6-2 (S))	Field evaluation without artificial inoculation (Vertisol and Inceptisol soils)	Katara et al. (2010)
2	***qBS2***	Hinohikari	110 RILs (Tadukan (R) and Hinohikari (S))	Inoculation of 18-day-old-plants in greenhouse	Sato et al. (2008a)
2	***BSq2.2v&i***	IR62266-42-6-2	154 DH lines (CT9993-5-10-1 M (R) and IR62266-42-6-2 (S))	Field evaluation without artificial inoculation (Vertisol and Inceptisol soils)	Katara et al. (2010)
4	***qBSfR4***	Hinohikari	110 RILs (Tadukan (R) and Hinohikari (S))	Field evaluation at 113 days after transplanting by promoting disease with inoculated spreader	Sato et al. (2015)
4	***BSq4.1v&i***	CT9993-5-10-1 M	154 DH lines (CT9993-5-10-1 M (R) and IR62266-42-6-2 (S))	Field evaluation without artificial inoculation (Vertisol and Inceptisol soils)	Katara et al. (2010)
6	*BSq6.1v*	CT9993-5-10-1 M	154 DH lines (CT9993-5-10-1 M (R) and IR62266-42-6-2 (S))	Field evaluation without artificial inoculation (Vertisol soil)	Katara et al. (2010)
6	*BSq6.2i*	CT9993-5-10-1 M	154 DH lines (CT9993-5-10-1 M (R) and IR62266-42-6-2 (S))	Field evaluation without artificial inoculation (Inceptisol soil)	Katara et al. (2010)
8	*BSq8.1i*	CT9993-5-10-1 M	154 DH lines (CT9993-5-10-1 M (R) and IR62266-42-6-2 (S))	Field evaluation without artificial inoculation (Inceptisol soil)	Katara et al. (2010)
8	*BSq8.2v*	CT9993-5-10-1 M	154 DH lines (CT9993-5-10-1 M (R) and IR62266-42-6-2 (S))	Field evaluation without artificial inoculation (Vertisol soil)	Katara et al. (2010)
9	***qBS9***	Tadukan	110 RILs (Tadukan (R) and Hinohikari (S))	Inoculation of 18-day-old-plants in greenhouse	Sato et al. (2008a)
9	**(QTL)**	Kasalath	39 CSSLs (Donor: Kasalath (R), Recipient: Koshihikari (S))	Inoculation of 18-day-old-plants in greenhouse	Sato et al. (2008b)
9	***BSq9.1v***	IR62266-42-6-2	154 DH lines (CT9993-5-10-1 M (R) and IR62266-42-6-2 (S))	Field evaluation without artificial inoculation (Vertisol soil)	Katara et al. (2010)
11	*BSq11.1v&i*	CT9993-5-10-1 M	154 DH lines (CT9993-5-10-1 M (R) and IR62266-42-6-2 (S))	Field evaluation without artificial inoculation (Vertisol and Inceptisol soils)	Katara et al. (2010)
11	***qBS11***	Tadukan	110 RILs (Tadukan (R) and Hinohikari (S))	Inoculation of 18-day-old-plants in greenhouse	Sato et al. (2008a)
11	***qBSfR11***	Tadukan	110 RILs (Tadukan (R) and Hinohikari (S))	Field evaluation at 113 days after transplanting by promoting disease with inoculated spreader	Sato et al. (2015)
11	***BSq11.2v***	IR62266-42-6-2	154 DH lines (CT9993-5-10-1 M (R) and IR62266-42-6-2 (S))	Field evaluation without artificial inoculation (Vertisol soil)	Katara et al. (2010)
12	*BSq12.1v*	CT9993-5-10-1 M	154 DH lines (CT9993-5-10-1 M (R) and IR62266-42-6-2 (S))	Field evaluation without artificial inoculation (Vertisol soil)	Katara et al. (2010)
12	*bs1*	Dinorado	186 F2 lines (Dinorado (R) and IR36 (S))	(not described in detail)	Banu et al. (2008)

^a^QTLs written in bold font were detected in similar positions in several cultivars in different experiments. QTLs with designations followed by v and/or i were identified on Vertisol and/or Inceptisol soil, respectively

^b^ *CSSLs* chromosome segment substitution lines, *DH* doubled haploid, *RIL* recombinant inbred lines, *R* resistant cultivar, *S* susceptible cultivar

To confirm the effects of these QTLs, advanced generations of the RILs and their parents were transplanted into a BS-infected paddy field and evaluated for resistance in 2012 and 2013. In that study, three QTLs for field resistance to BS (*qBSfR1*, *qBSfR4* and *qBSfR11*) were detected (Sato et al. 2015). The ‘Tadukan’ alleles at *qBSfR1* and *qBSfR11* and the ‘Hinohikari’ allele at *qBSfR4* enhanced resistance to BS. The QTL with the largest effect, *qBSfR11*, explained 17.9 and 19.2 % of the phenotypic variation in 2012 and 2013, respectively, and was thought to coincide with *qBS11*, which had been detected at the seedling stage (Sato et al. 2008a). Therefore, the resistance derived from *qBSfR11* (*qBS11*) was consistently detectable regardless of growth stages. To verify the resistance conferred by the ‘Tadukan’ alleles at *qBSfR1* and *qBSfR11*, near-isogenic lines (NILs) containing each of these QTL alleles from ‘Tadukan’ in the background of susceptible variety ‘Koshihikari’ were bred and evaluated under field conditions. A NIL containing the ‘Tadukan’ allele at *qBSfR11* acquired significant BS field resistance, but that with the ‘Tadukan’ allele at *qBSfR1* did not. Besides ‘Tadukan’, ‘Kasalath’ was also identified as a resistant cultivar (Sato et al. unpublished data). By using chromosome segment substitution lines (CSSLs) derived from a cross between ‘Kasalath’ and ‘Koshihikari’, Sato et al. (2008b) detected one QTL located on chromosome 9 near *qBS9*.

Because soil fertility is known to affect BS levels, Katara et al. (2010) searched for QTLs associated with field resistance to BS in rice grown on two different soil types: Vertisol and Inceptisol. According to the report, Vertisol is easily recognized because of its clayey texture, dark color, and unique physical characteristics and is very productive if well managed, but presents challenges to low-input agriculture (Katara et al. 2010). Inceptisols are mineral soils that have significant subsoil or surface alteration. Using doubled haploid (DH) lines derived from a cross between ‘CT 9993-5-10-1-M’ (resistant) and ‘IR 62266-42-6-2’ (susceptible), a total of 11 QTLs were detected, four of which (*BSq2.1v&i*, *BSq2.2v&i*, *BSq4.1v&i* and *BSq11.1v&i*) were detected in tests on both soil types (Katara et al. 2010). *BSq4.1v&i* and *BSq11.1v&i*, which are located on chromosomes 4 and 11, respectively, explained the highest percentages of phenotypic variation. *BSq4.1v&i* explained 18.5 % of the total phenotypic variation when rice was grown on Vertisol and 22.3 % when grown on Inceptisol. *BSq11.1v&i* explained 13.1 % of the total phenotypic variation when rice was grown on Vertisol and 12.9 % when grown on Inceptisol. Comparing the QTLs detected by Sato et al. (2008a, b) with those of Katara et al. (2010), four chromosomal regions may be common to both studies: (1) *qBS2* and *BSq2.2 v&i*, (2) *qBSfR4* and *BSq4.1 v&i*, (3) *qBS9,* unnamed QTL reported in Sato et al. (2008b) and *BSq9.1v*, and (4) *qBS11*, *qBSfR11*, and *BSq11.2v*.

Two QTLs were identified from F_2_ lines derived from a cross between landrace ‘Dinorado’ (resistant) and ‘IR36’ (susceptible) and *bs1* was located on chromosome 12 (Banu et al. 2008). On the basis of map information, *bs1* seems to be different from *BSq12.1v* (Katara et al. 2010; Leung et al. 2015). By a follow-up study, *bs1* with significant SNP markers spanning a 1.3 Mb region was identified (Leung et al. 2015). With this region, local haplotypes, consisting of 7 SNPs were associated with resistance and the ‘resistant’ haplotype was present in 2777 (93 %) of the 3000 accessions (Leung et al. 2015). According to the report, a possible explanation is that landraces or traditional varieties were selected for disease resistance (BS) or other traits tightly linked to this region by farmers long ago before modern rice breeding (Leung et al. 2015).

### Screening cultivars for resistance to bacterial seedling rot (BSR)

Although the same organism (*Burkholderia glumae*) causes both BSR and BGR in rice, the number of reports of screening cultivars for resistance to BSR is smaller than that for BGR (Table 3). Goto (1982) developed an evaluation method for BSR in which a bacterial suspension is injected into the soil at germination. A total of 293 cultivars and breeding lines were screened in Japan using this method and variation in resistance level was observed, although no highly resistant cultivars or lines were found (Goto 1983a). Another study was conducted in Japan in which 17 cultivars were screened by inoculation of sterilized seeds, and ‘Kujuu’ was found to be resistant (Hirashima and Wakimoto 1983).

**Table 3:** Previous reports of screening cultivars and breeding lines for resistance to bacterial seedling rot (BSR)

Year	Country^a^	Resistant cultivars and breeding lines	Number of screened cultivars or lines	Evaluation method	Reference(s)
1983	Japan	(Resistance levels differed among the materials, although no highly resistant cultivars/lines were found.)	293	Injection of bacterial suspension into soil at germination	Goto (1982, 1983a)
1983	Japan	Kujuu	17	Inoculation of sterilized seeds	Hirashima and Wakimoto (1983)
2006	USA	LM-1, Drew	6	Inoculation of 28-day-old-plants	Sayler et al. (2006)
2014	USA	(Lesion sizes were too inconsistent to be used to differentiate resistance levels among cultivars.)	300	Seedlings were needle inoculated.	Wamishe et al. (2014)

^a^Country where experiment was conducted

In recent years, damage by *Burkholderia glumae* has also become severe in the United States (Ham et al. 2010). In 2006, Sayler and colleagues inoculated 28-day-old plants and found that ‘LM-1’ and ‘Drew’ exhibited resistance to BSR based on disease lesion size and bacterial growth *in planta* (Sayler et al. 2006). In 2014, Wamishe and colleagues analyzed BSR resistance of 300 cultivars by needle inoculation of seedling leaves and found that lesion sizes were too inconsistent to be used to differentiate resistance levels among cultivars (Wamishe et al. 2014). Differences in evaluation methods could be the reason why common resistant cultivars were not detected across the reports summarized in Table 3.

### Screening cultivars for resistance to bacterial grain rot (BGR)

The severity of BGR infection is affected by host susceptibility, inoculum density, humidity, and temperature (Goto 1983b; Tsushima 1996). In Japan, damage by BGR was often observed in southern areas until oxolinic acid was developed as an agricultural chemical for BGR control (Hikichi et al. 1989; Hikichi 1993a, b). In field observations over 7 years, six cultivars were found to be relatively resistant (Yokoyama and Okuhara 1987) (Table 4). By a different research group of 85 cultivars over 3 years, three showed moderate resistance in field observations (Yasunaga et al. 2002) (Table 4). In 1975, Goto and Watanabe analyzed nine cultivars by field evaluation of plants inoculated at the flowering stage. They found that none showed complete resistance and classified the cultivars as relatively resistant, moderate, or susceptible (Goto and Watanabe 1975). Among them, three cultivars were reported to be relatively resistant. Later, Goto developed an evaluation method in which large numbers of plants were cultivated in the greenhouse and inoculated at the flowering stage (Goto 1982). Using this method, Goto analyzed 293 cultivars and breeding lines and found that resistance levels differed among the materials, although no highly resistant cultivars or lines were identified (Goto 1983a). Similar results were reported by other groups (Mogi and Tsushima 1985; Takita et al. 1988). In field evaluation at the flowering stage, Imbe and colleagues found that breeding line ‘Saikai169’ was more susceptible than ‘Saikai170’ even though both were derived from the same cross (Imbe et al. 1986). The authors speculated that resistance to BGR was genetically controlled. In a greenhouse screen of 14 wild rice species inoculated at flowering stage, 10 accessions (*O. meridionalis*, *O. stapfii*, *O. australiensis*, *O. officinalis*, *O. grandiglumis*, *O. glaberrima* (OG-2), *O. rufipogon*, *O. longistaminata*, *O. punctata*, and *O. alta*) were found to be resistant (Tsushima et al. 1989).

**Table 4:** Previous reports of screening cultivars and breeding lines for resistance to bacterial grain rot (BGR)

Year	Country^a^	Resistant cultivars and breeding lines	Number of screened cultivars or lines	Evaluation method	Reference(s)
1975	Japan	Fukumasari, Tachikaze, Kinmaze	9	Field evaluation (inoculation at flowering stage)	Goto and Watanabe (1975)
1983	Japan	(Resistance levels differed among the materials, although no highly resistant cultivars/lines were found.)	293	Greenhouse evaluation (inoculation at flowering stage)	Goto (1982, 1983a)
1985	Japan	(No source of complete resistance was identified.)	11	Field evaluation (inoculation at flowering stage)	Mogi and Tsushima (1985)
1986	Japan	(Saikai169 identified as a susceptilble line)	6	Field evaluation (inoculation at flowering stage)	Imbe et al. (1986)
1987	Japan	Nihonmasari, Koganebare, Mineasahi, Toyotama, Nishihomare, Kougyoku	20	Field observation without artificial inoculation over 7 years	Yokoyama and Okuhara (1987)
1987	China	KaohsiungS.7	21	Field evaluation (inoculation before flowering stage)	Chien and Chang (1987)
1988	Japan	(Resistance levels differed between cultivars, although no highly resistant cultivars were found.)	427	Field evaluation (inoculation at flowering stage)	Takita et al. (1988)
1988	Brazil	Limeira, Iguape Redondo	19	Field Evaluation (inoculation of bacterial suspension into boots by syringe)	Prabhu and Bedendo (1988)
1989	Japan	*O. meridionalis, O. stapfii, O. australiensis, O. officinalis, O. grandiglumis, O. glaberrima (OG-2), O. rufipogon, O. longistaminata, O. punctata, O. alta*	14	Evaluation of wild rice accessions in greenhouse (inoculation at flowering stage)	Tsushima et al. (1989)
1989	Japan	Akuranboda, Col 155	22	Field evaluation (inoculation at flowering stage) and cut-panicle inoculation	Miyagawa and Kimura (1989)
1994	Japan	Resistant Japanese cultivars: Sasanishiki, Kokuryoumiyako, Benisengoku, Jukkoku, Mizuho, Kogyoku	129	Field Evaluation (inoculation of bacterial suspension into boots by syringe)	Wasano and Okuda (1994)
		Resistant non-Japanese cultivars: Palkeng, Century Patna, Belle Patna 9433, Hybrid Pearl, Blue Bonnet 50, RD-23			
2002	Japan	Chikushi52, Chikushi41, Tsukushiwase	85	Field observation without artificial inoculation over 3 years	Yasunaga et al. (2002)
2007	USA	Jupiter	5	Field evaluation (inoculation at flowering stage)	Nandakumar et al. (2007a)
2007	USA	LM-1, LMT-1	(2)^b^	Field evaluation (inoculation at flowering stage)	Groth et al. (2007)
2013	Japan	Kele, Kasalath, Jhona2, Jaguary, Khau Mac Kho	84	Cut-panicle inoculation	Mizobuchi et al. (2013a)
2014	USA	14 lines were identified as resistant, 30 lines as moderately resistant (2012)	300	Field evaluation (inoculation from boot split to flowering stage) over 2 years	Wamishe et al. (2014)
		15 lines were identified as resistant, 53 lines as moderately resistant (2013)			

^a^Country where experiment was conducted

^b^LM-1 and LMT-1 were selected from 1 kg of mutagenized seeds of Lemont irradiated with gamma radiation (250 Gy) from 60Co

Although most evaluation for BGR resistance has involved inoculation at the flowering stage, other evaluation methods have been developed. One method is field evaluation by inoculation of bacterial suspension into the boots (i.e., onto panicles prior to emergence from the stem) with a syringe (Prabhu and Bedendo 1988; Wasano and Okuda 1994). By this method, six Japanese cultivars and six cultivars from other countries were found to be resistant (Wasano and Okuda 1994), and in Brazil, cultivars ‘Limeira’ and ‘Iguape Redondo’ were found to be resistant (Prabhu and Bedendo 1988). Yet another method of evaluation is cut-panicle inoculation, which is based on the ‘cut-spike’ test developed for the evaluation of *Fusarium* head blight resistance in barley (Takeda and Heta 1989; Hori et al. 2005). To minimize environmental variation at the time of inoculation, this method entails the collection of panicles from field-grown plants and their inoculation under controlled conditions at the time of flowering (Miyagawa and Kimura 1989). Because the correlation coefficient between the disease rating obtained by cut-panicle inoculation and that obtained by pot inoculation is very high (*r* = 0.868), cut-panicle inoculation was recognized as a useful method for evaluating BGR resistance, and two out of 22 cultivars screened were found to be resistant (Miyagawa and Kimura 1989). By using a cut-panicle inoculation method, Mizobuchi and colleagues evaluated 84 cultivars, including 62 accessions from WRC [World Rice Collection of the National Institute of Agrobiological Sciences (NIAS) (Kojima et al. 2005)], and five cultivars including ‘Kele’ were found to be relatively resistant to BGR (Mizobuchi et al. 2013a).

In addition to the work on BGR in Japan, reports from other countries are increasing. All of the evaluations summarized here involved inoculation of field-grown plants at or before flowering. In China, ‘Kaohsiung S.7’ was found to be resistant (Chien and Chang 1987). In the United States, ‘Jupiter’ (Nandakumar et al. 2007a) and two other lines (Groth et al. 2007) were identified as resistant; the latter two lines were selected from mutagenized seeds of ‘Lemont’ irradiated with gamma radiation (250 Gy) from Cobalt-60. Recently in the United States, Wamishe and colleagues analyzed 300 entries in the Uniform Regional Rice Nursery (URRN) and Arkansas Rice Performance Trials (ARPT) over 2 years; in these tests, over 10 entries showed resistance and over 30 entries showed moderate levels of resistance (Wamishe et al. 2014).

### Analysis of QTLs for resistance to bacterial seedling rot (BSR) and bacterial grain rot (BGR)

Although *Burkholderia glumae* causes both BSR and BGR, no correlation between resistance to the two diseases was observed in an early study (Goto 1983a). Similarly, cultivars partially resistant to BGR (Mizobuchi et al. 2013a) were not always resistant to BSR (Mizobuchi et al. in preparation). Therefore, the molecular mechanisms for resistance to BSR appear to be different from those for resistance to BGR.

To date, only one QTL for resistance to BSR has been identified (Mizobuchi et al. 2013b). This QTL (*qRBS1*, *quantitative trait locus to resistance to bacterial seedling rot 1*) was found on chromosome 10 by using CSSLs derived from a cross between ‘Nona Bokra’ (resistant) and ‘Koshihikari’ (susceptible) (Table 5). *qRBS1* was mapped in the region of *RM24930*–*RM24944* and located by substitution mapping in a 393-kb interval of the short arm of chromosome 10 in the ‘Nipponbare’ genome reference sequence (Mizobuchi et al. 2013b). This QTL explained 22 % of the total phenotypic variation for resistance to BSR in an F_5_ population derived from a cross between a resistant CSSL and ‘Koshihikari’, and was thought to be involved in a key process for resistance to *Burkholderia glumae* at the seedling stage. Because *qRBS1* is the first fine-mapped QTL related to resistance to *Burkholderia glumae*, it was renamed as *RBG1* (*Resistance to Burkholderia glumae 1*) (Mizobuchi et al. 2013b; Mizobuchi et al. 2015). According to the QTL Annotation Rice Online Database (Q-TARO) (Yonemaru et al. 2010), no QTLs related to disease resistance have been found in the region of *RM24930*–*RM24944*. Therefore, *RBG1* appears to be a novel QTL.

**Table 5:** QTLs for resistance to bacterial seedling rot (BSR) and bacterial grain rot (BGR)

Chromosome	QTL	Source of resistance allele	Materials used for QTL analysis^a^	Phenotyping method	Reference(s)
Resistance to BSR					
10	*RBG1 (qRBS1)*	Nona Bokra	44 CSSLs (Donor: Nona Bokra (R), Recipient: Koshihikari (S))	Inoculation of sterilized seeds	Mizobuchi et al. (2013b)
Resistance to BGR					
1	*RBG2*	Kele	110 BILs (Kele (R) and Hitomebore (S))	Modified cut-panicle inoculation^b^	Mizobuchi et al. (2013a, 2015)
1	*qBPB-1-1*	TeQing	300 RILs (TeQing (R) and Lemont (S))	Field evaluation (inoculation from full boot stage to flowering stage)	Pinson et al. (2010)
1	*qBPB-1-2*	TeQing	300 RILs (TeQing (R) and Lemont (S))	Field evaluation (inoculation from full boot stage to flowering stage)	Pinson et al. (2010)
1	*qBPB1-3*	Lemont	300 RILs (TeQing (R) and Lemont (S))	Field evaluation (inoculation from full boot stage to flowering stage)	Pinson et al. (2010)
2	*qBPB-2-1*	Lemont	300 RILs (TeQing (R) and Lemont (S))	Field evaluation (inoculation from full boot stage to flowering stage)	Pinson et al. (2010)
2	*qBPB-2-2*	TeQing	300 RILs (TeQing (R) and Lemont (S))	Field evaluation (inoculation from full boot stage to flowering stage)	Pinson et al. (2010)
3	*qBPB-3-1*	TeQing	300 RILs (TeQing (R) and Lemont (S))	Field evaluation (inoculation from full boot stage to flowering stage)	Pinson et al. (2010)
3	*qBPB-3-2*	Lemont	300 RILs (TeQing (R) and Lemont (S))	Field evaluation (inoculation from full boot stage to flowering stage)	Pinson et al. (2010)
7	*qBPB-7*	TeQing	300 RILs (TeQing (R) and Lemont (S))	Field evaluation (inoculation from full boot stage to flowering stage)	Pinson et al. (2010)
8	*qBPB-8-1*	TeQing	300 RILs (TeQing (R) and Lemont (S))	Field evaluation (inoculation from full boot stage to flowering stage)	Pinson et al. (2010)
8	*qBPB-8-2*	Lemont	300 RILs (TeQing (R) and Lemont (S))	Field evaluation (inoculation from full boot stage to flowering stage)	Pinson et al. (2010)
10	*qBPB-10*	TeQing	300 RILs (TeQing (R) and Lemont (S))	Field evaluation (inoculation from full boot stage to flowering stage)	Pinson et al. (2010)
11	*qBPB-11*	TeQing	300 RILs (TeQing (R) and Lemont (S))	Field evaluation (inoculation from full boot stage to flowering stage)	Pinson et al. (2010)

^a^ *BILs* backcross inbred lines, *CSSLs* chromosome segment substitution lines, *RILs* recombinant inbred lines, *R* resistant cultivar, *S* susceptible cultivar

^b^Spikelets were inoculated 1 day after anthesis

On the other hand, a large number of QTLs for resistance to BGR have been identified (Table 5). (Pinson et al. 2010) used a set of RILs derived from a cross between ‘TeQing’ (resistant) and ‘Lemont’ (susceptible) to identify QTLs conferring resistance to BGR at the flowering stage in 2001 and 2002. The evaluation method involved repeated inoculation of panicles in the field from full boot stage to flowering stage. Using these RILs, 12 QTLs were identified on seven chromosomes (Table 5). Among these QTLs, three (*qBPB-1-3*, *qBPB-3-1* and *qBPB-3-2*) were statistically significant in both years. The favorable alleles for eight of the QTLs were derived from ‘TeQing’; those for the other four QTLs were derived from ‘Lemont’. Four QTLs were co-located with QTLs previously associated with resistance to other diseases: *qBPB-2-1* was mapped near a QTL for resistance to sheath blight (Pinson et al. 2005), *qBPB-2-2* was mapped near QTLs for resistance to blast (Tabien et al. 2002) and bacterial leaf blight (Li et al. 1999), *qBPB-3-1* was mapped near QTLs for resistance to blast and sheath blight, and *qBPB-8-2* was mapped near QTLs for resistance to sheath blight and bacterial leaf blight. A major QTL, *qBPB-3-1*, which accounted for approximately 14 % of phenotypic variation in the mean of the 2001 and 2002 annual averages, was found to be co-located with a QTL for heading date. Two other QTLs (*qBPB-8-2* and *qBPB-10*) were also co-located with QTLs for late flowering. Because late-flowering panicles are subjected to cooler temperatures that are less conductive to disease development during grain fill, it is possible that the genetic effects of the heading-related QTLs affected the disease scoring. Pinson et al. (2010) concluded that the data could not distinguish between pleiotropy and close linkage of the QTLs identified previously.

In another study, a major QTL for BGR resistance was mapped on the long arm of chromosome 1 using backcross inbred lines (BILs) derived from a cross between ‘Kele’ (resistant) and ‘Hitomebore’ (susceptible) (Mizobuchi et al. 2013a). ‘Kele’ and ‘Hitomebore’ were selected for BIL development and genetic analysis after prescreening of 84 cultivars. A modified cut-panicle inoculation method was applied to minimize environmental effects: specifically, panicles containing only spikelets at one day after anthesis were harvested and inoculated. To search for QTLs associated with BGR resistance, the ratio of diseased spikelets (RDS, an index reflecting both quantity and severity of infection) and the ratio of diseased spikelet area (RDSA) were measured. In the BILs, the detected QTL explained 25.7 % and 12.1 % of the total phenotypic variation in RDS and RDSA, respectively, and the ‘Kele’ allele increased BGR resistance. On the other hand, no QTLs for agronomic traits such as culm length, panicle length, panicle number, spikelet length, spikelet width or heading date were detected close to the QTL for RDS and RDSA. In a follow-up study (Mizobuchi et al. 2015), substitution mapping using homozygous recombinant and nonrecombinant plants demonstrated that the QTL, designated as *RBG2*, was located in a 502-kb interval defined by *RM1216* and *RM11727* on the long arm of chromosome 1. On the basis of map information, *RBG1* seems to be different from *qBPB-10*, and *RBG*2 seems to be different from *qBPB-1-1*, *qBPB-1-2* and *qBPB-1-3*.

## Conclusions

As reviewed here, several QTLs for resistance to BS, BSR and BGR have been detected owing to improvements in evaluation methods for disease resistance and to recent progress in rice molecular genetics. As an example of the former, a seedling evaluation method for BS resistance was developed that enabled detection of several QTLs, including *qBS11* (Sato et al. 2008a; Sato et al. 2008b). In a later study, *qBS11* was reconfirmed as *qBSfR11*, a QTL for field resistance to BS (Sato et al. 2015). *RBG2*, which confers resistance to BGR, was identified by a modified cut-panicle inoculation method (Mizobuchi et al. 2013a) that minimizes environmental influences such as humidity and temperature. Importantly, no QTL alleles for agronomic traits such as heading date were linked to *RBG2*, suggesting that the effects of this locus might be detectable regardless of environmental conditions or other agronomic traits. It will be necessary to confirm the effectiveness of *RBG2* under field conditions in order to use it for breeding resistant cultivars.

Advanced-backcross progeny such as CSSLs can be very useful for genetic analysis (Ebitani et al. 2005; Takai et al. 2007; Fukuoka et al. 2010a; Fukuoka et al. 2010b). Because each CSSL has only one or a few segment substitutions, it is possible to detect QTLs with minor effects contained within the substituted segments. By using CSSLs, *RBG1* (resistance to BGR) and a QTL for BS resistance were detected (Sato et al. 2008b; Mizobuchi et al. 2013b).

Recent progress in genomics has enhanced understanding of the genetic basis of agronomic traits, and development of crops with the desired traits could enhance adaptation to climate change or mitigate its effects (Yamamoto et al. 2009; Abberton et al. 2016; Kole et al. 2015). For example, genome-wide identification of single-nucleotide polymorphisms between BGR-resistant cultivar ‘Jupiter’ (Nandakumar et al. 2007a) and a susceptible control cultivar has been conducted as a step toward fine mapping the resistance in this line (Shrestha et al. 2014). Although several QTLs have been fine mapped, none have yet been cloned, so the mechanisms underlying the QTLs reviewed in this paper are still unknown. Isolation and characterization of *RBG1* (for resistance to BSR) is now underway (Mizobuchi et al. unpublished data). Isolation of *RBG1* and other genes underlying disease resistance QTLs will elucidate the genetic mechanisms of resistance.

The race specificity of the host resistance to BS, BSR and BGR is critical for the utilization of resistance QTLs in varietal improvement. By a screen of 80 cultivars in 1974, eight cultivars including ‘Tadukan’ were found to be resistant to many strains of BS (Ohata and Kubo 1974; Ohata 1989). In a later study, *qBS11* from Tadukan was reconfirmed as *qBSfR11*, a QTL for field resistance to BS (Sato et al. 2008a; Sato et al. 2015). *qBS11* was found by inoculation of a strain (D6-2), while a different stain was isolated from the field in the research of *qBSfR11*. Therefore *qBSfR11* seems to be a QTL without race-specificity. NIL harboring *RBG1* (for resistance to BSR) seems to be multiple resistance to several strains, indicating *RBG1* is a QTL without race-specificity (Mizobuchi et al. unpublished data). On the other hand, the presence of the race specificity of *RBG2* (for resistance to BGR) is unknown.

Although BSR and BGR are caused by the same pathogen (*Burkholderia glumae*), no cultivars were found as resistance to BSR and BGR. However, the number of reports of screening cultivars for resistance to BSR is much smaller than that for BGR. Thus the correlation of *RBG* in seedling and panicle stages could be under-estimated. Characterization of *RBG1* and *RBG2* is now underway (Mizobuchi et al. unpublished data).

Is it possible to breed ‘ideal’ resistant cultivars by using only the QTLs described in this paper? Of course, the answer is no. Although several major QTLs have been detected—*qBS9* and *BSq9.1, qBS11* and *qBSfR11* for BS, *RBG1* for BSR and *RBG2* for BGR—none of them explained over 30 % of the phenotypic variation in the QTL analysis. Thus, it will be necessary to identify new QTLs from different sources for gene pyramiding. A recent study (Fukuoka et al. 2015) confirmed that gene pyramiding enhances durable blast disease resistance in rice. In addition, the factors associated with resistance to BSR and BGR seem to be different, so it will be necessary to combine *RBG1*, *RBG2* and additional QTLs for stable resistance to both diseases. Because BS, BSR and BGR are common diseases that have been increased by global warming, the final goal is to pyramid QTLs for resistance into elite germplasm as a means of developing cultivars with resistance to all of these important diseases.
